# Efficient Prestress Wedge Flaw Detection Using a Lightweight Computational Framework

**DOI:** 10.3390/s25226978

**Published:** 2025-11-14

**Authors:** Qingyu Yao, Yulong Guo, Weidong Liu

**Affiliations:** 1 Faculty of Engineering, Huanghe Science and Technology University, Zhengzhou 450003, China; 2 School of Resources and Environment, Henan Agricultural University, Zhengzhou 450002, China; gyl.zh@henau.edu.cn; 3 Dr Aha Data and AI Technology Pty. Ltd., Sydney 2118, Australia; weidongliu@drahadataai.com

**Keywords:** prestress, wedges, detection, FasterNET-YOLOv5, efficiency, robust

## Abstract

Prestressing wedges are critical in bridge and road construction, but flaws in wedge threads lead to severe safety hazards, construction delays, and costly maintenance. Traditional manual inspection remains labor-intensive and inconsistent, particularly under variable illumination and complex surface conditions. However, few studies have investigated improving the inspection effectiveness. Therefore, this study aims to propose a lightweight FasterNET-YOLOv5 framework for accurate and robust prestress wedge flaw detection in industrial applications. The framework achieves a detection precision of 96.3%, recall of 96.2, and mAP@0.5 of 96.5 with 18% faster end-to-end inference speed, enabling deployable system configuration on portable or embedded devices, making the approach suitable for real-time industrial inspection. Further practical guidance for workshop inspection illumination conditions was confirmed by robustness evaluations, as white lighting background provides the most balanced performance for incomplete thread and scratch defects. Moreover, a mechanical model-based inverse method was exploited to link the detections from machine vision. The results also demonstrate the potential for broader 3D surface inspection tasks in threaded, machined, and curved components of intelligent, automated, and cost-effective quality control. In general, this research contributes to computational inspection systems by bridging deep learning-based flaw detection with engineering-grade reliability and deployment feasibility.

## 1. Introduction

**Figure 1:**
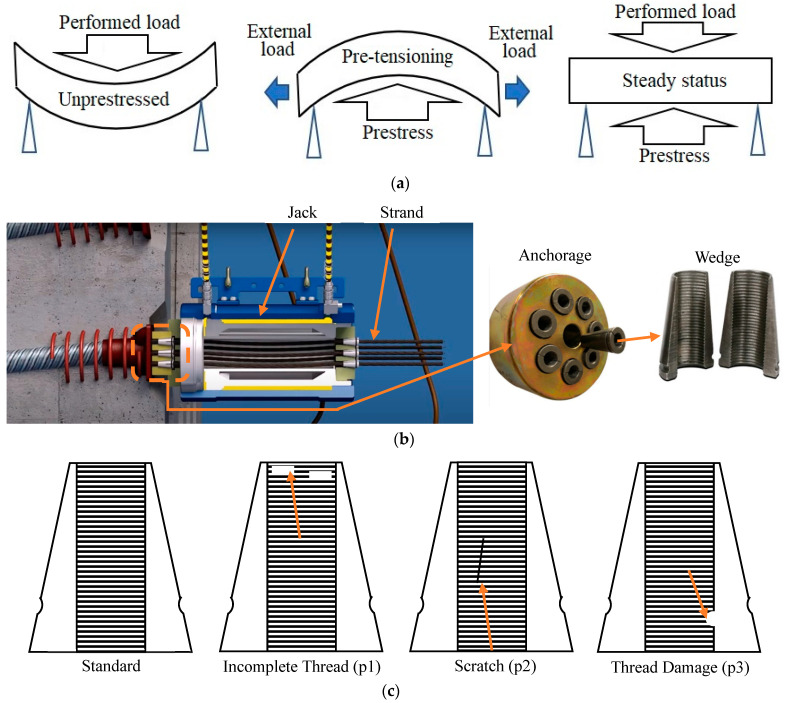
(**a**) Schematic process of prestressing; (**b**) schematic setup of anchorage pre-tensioning system; (**c**) wedge flaws.

Prestress technology is widely applied in large-scale civil infrastructure, including bridges, highways, and railways, to enhance structural capacity and durability [1,2,3,4,5]. By inducing internal compressive stresses, prestressing effectively counteracts tensile loads and reduces cracking, thereby extending service life, as shown in Figure 1a. Then, a central element of the system is the anchorage assembly (Figure 1b), where wedges grip strands under high tension. The reliability of wedge threads directly determines the safety of prestressing operations: defects such as incomplete threads, scratches, or localized wear (Figure 1c) can cause slippage or fracture, leading to catastrophic strand release and structural failure [6]. Given that each prestressed structure may require thousands of wedges, ensuring the integrity of these small but critical components is of urgent industrial importance.

Existing inspection practices, such as manual visual examination and ultrasonic testing, are inefficient, inconsistent, and highly sensitive to environmental conditions [6,7,8,9,10,11], which are unsuitable for large-scale deployment on construction sites or production lines, where rapid and standardized quality assurance is essential. Moreover, wedge flaws present unique challenges compared to flat-surface materials, three-dimensional geometry, and fine-scale thread details, and reflective metallic surfaces complicate defect recognition, particularly under mixed illumination. These factors highlight the need for intelligent, automated inspection systems capable of handling real-world variability while maintaining accuracy, speed, and portability.

With advances in artificial intelligence, computer vision and deep learning-based methods have emerged as powerful alternatives. Convolutional neural networks (CNNs) have succeeded in surface flaw detection [12,13], weld defect identification [14], and vibration-based damage monitoring [15]. Object detection frameworks like Faster R-CNN [16] and the YOLO family [17,18] are widely adopted for balancing accuracy and efficiency. Benefited by user-friendly modularity, ease of training, and robustness across scenarios [19], YOLOv5 stands out and is widely exploited in industrial inspection [20]. Nevertheless, YOLOv5’s CSPDarknet53 backbone is computationally heavy, limiting the deployment on embedded/edge devices for real-time industrial use [21]. Recent advances in YOLO-based industrial defect detection have focused on balancing accuracy, efficiency, and scenario adaptability via modular upgrades and lightweight designs. An improved YOLOv8 for automotive surface defects adopts lightweight components and weighted feature fusion, cutting parameters by 36.62% and FLOPs by 37.28% while boosting mAP by 3.5% on the custom Auto-DET dataset [22]. For transmission line component detection, GMPPD-YOLO (a YOLOv5 variant) integrates grouped dense feature extraction, multi-scale pooling, and partial convolution, plus pruning and distillation—achieving 68.4% fewer parameters, 58.2% lower FLOPs, and 1%+ gains in precision and mAP50 [23]. In steel defect detection, SCCI-YOLO (YOLOv8n-based) uses SPD-Conv and cross-scale fusion to reduce parameters by 43.9% and reach 78.6% mAP on NEU-DET [24]; EAD-YOLOv10 (YOLOv10-based) leverages adaptive downsampling and dynamic upsampling, achieving 94.2% mAP on NEU-DET (7.6% above baseline) with a 9.75% smaller model size [25]. These variants excel in general industrial defects but lack optimization for 3D threaded geometries (e.g., prestress wedge threads), full adaptability to low-cost edge devices, and robustness to variable industrial illumination. Notably, one of the recent lightweight backbones, FasterNET, exerts partial convolution to reduce redundant computations for feature extraction, at an effective balance of achieving superior computational efficiency while maintaining effective feature representation [19,26].

Despite the rapid development of deep learning applications, few studies were recorded on wedge flaw inspections: Xiao et al. [27] exploited the gray-scale distribution characteristics of wedge images to approximately determine the defect types. In 2019, Tang [28] further proposed a focal loss-based residual network to identify the thread defects. However, feature extraction for improved defect classifications became a necessity, and the efficiency of real-time recognition still needs to be further optimized. Significantly, products’ complex geometries and 3D surface textures induce intricate reflections, insensitive occlusions, and fine-scale defects, which are not common in flat or uniform materials [19,28]. Hence, this research aims at replacing YOLOv5 backbone with FasterNET to evaluate the applicability of a merged and more efficient FasterNET-YOLOv5 framework for the new application of promising robust, real-time wedge flaw detection under mixed lighting resources in future prestressing industrial practices. From a computational engineering perspective, the proposed work represents a deployable inspection pipeline integrating model compression, real-time computation, and data visualization for structural integrity assessment, contributing to the development of a reproducible, low-cost, and modular software system and enabling intelligent inspection in engineering applications.

## 2. Methodology

To address the core industrial challenges of prestress wedge inspection, such as limited on-site computational resources, real-time detection requirements for production lines, and reliable identification of safety-critical flaws, an engineering design logic of replacing YOLOv5 CSPDarknet53 backbone with FasterNET was discussed, with all technical choices tied to practical industrial deployment needs.

### 2.1. The Core of FasterNET

FasterNET was selected as the alternative backbone primarily for its inherent adaptability to industrial constraints. The core partial convolution (PConv) strategy processes only a subset of input channels instead of all, drastically reducing memory occupation and computational demand. It is critical for deployment on low-cost edge devices that small-to-medium prestress component factories can afford. Specifically, FasterNET exploits a grouping parameter *P* to dynamically adjust channels. The quantitative demonstrations for reductions in parameters and floating-point operations (FLOPs) can be shown in Equations (1) and (2).

(1) $${\mathrm{Parameters}}_{\mathrm{partial}}=P\times k^{2}\times \frac{C_{in}}{P}\times \frac{C_{out}}{P}=\frac{k^{2}\times C_{in}\times C_{out}}{P}$$

(2) $${\mathrm{Flops}}_{\mathrm{partial}}=P\times \left(k^{2}\cdot \frac{C_{in}}{P}\cdot \frac{C_{out}}{P}\cdot H\cdot W\right)=\frac{k^{2}\times C_{in}\times C_{out}}{P}\cdot H\cdot W$$

where $C_{in}$ and $C_{out}$ denote the input and output channels, respectively, H and W are the height and width of output features, and k represents the kernel size with a common value of 3. Notably, the parameters and Flops from traditional convolution are shrunk by grouping number *P*. Furthermore, shallow layers, capturing low-level and redundant thread contours, adopt *P* = 4 for aggressive computation reduction, while deep layers, distinguishing subtle flaws like shallow scratches or localized wear, use *P* = 2 to maximally retain critical defect features [26]. And these operations ensure no safety hazards arise from missed detections, aligning with the strict reliability requirements of prestress engineering.

### 2.2. Structure of YOLOv5

**Figure 2:**
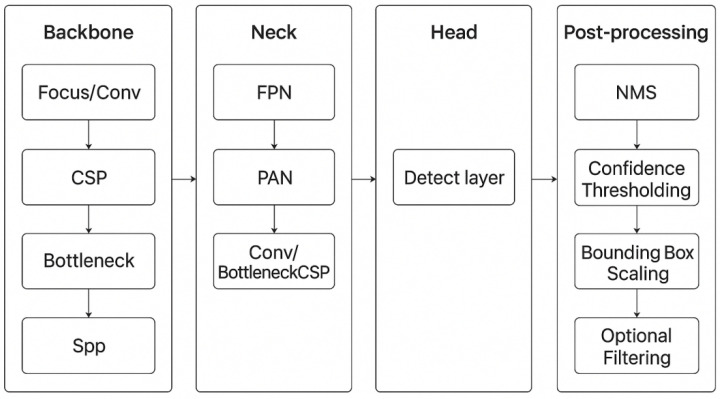
A brief flow chart of YOLOv5.

YOLOv5 (v6.0) served as the basic framework for industrial-grade modularity, capable of backbone replacement without disrupting the entire detection workflow, which is a key advantage for factory engineers to integrate the model into existing production lines. There are four core modules, as shown in Figure 2, inherently aligned with wedge inspection needs, reducing the need for large-scale overhauls of existing industrial systems. The original CSPDarknet53 backbone provides a proven feature extraction foundation, though heavy for on-site use. The Neck, consisting of FPN (Feature Pyramid Network) and PANet (Path Aggregation Network), fuses multi-scale features to detect non-uniform flaws, from large thread gaps to small scratches. The head module takes the fused features to map the channels to the prediction dimensions, output raw flaw prediction feature maps in training mode, and decode coordinates to generate candidate bounding boxes in inference mode. The post-processing module, which is made up of confidence thresholding and NMS (Non-Maximum Suppression), filters false positives to avoid unnecessary production line downtime.

### 2.3. The Integration of FasterNET

**Figure 3:**
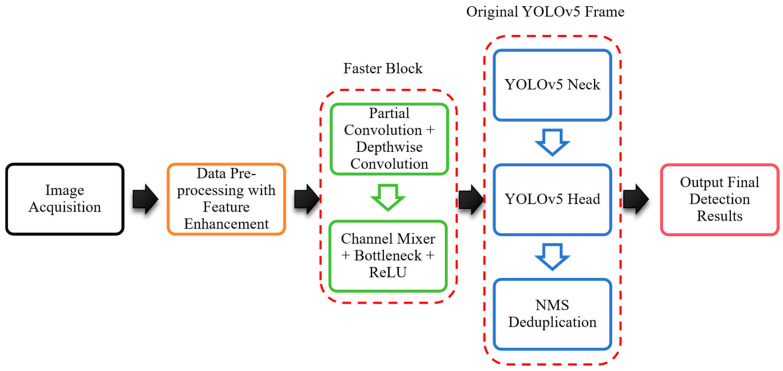
Workflow of FasterNET-YOLOv5 framework.

The implementation of replacing CSPDarknet53 with FasterNET (pre-trained weight file FasterNET_t0-epoch.281-val_acc1.71.9180.pth) focused on minimizing modification costs and maximizing compatibility. First, a dedicated branch was added to the parse_model function in yolo.py file to recognize FasterNET blocks, enabling compatibility with industrial-oriented pre-trained weights. Channel record lists and layer index offsets were adjusted to ensure FasterNET outputs 3-scale features (1/8, 1/16, 1/32) to match the requirements of Neck input, avoiding costly redesigns of downstream modules. The _forward_once function was also optimized to cache intermediate features correctly, preserving real-time performance for conveyor line speeds. Then, a simplified YAML configuration file was customized to lower deployment barriers for edge devices, and the width_multiple parameter was set to align FasterNET output channels with the original Neck. A whole work flow for the FasterNET-YOLOv5 frame is shown in Figure 3. Partial convolution is applied to partial input channels for spatial feature extraction, via 5-stage channel variation, 3-32-64-128-256-512. Consequently, the FLOPs and memory access volume of PConv are significantly reduced; the specific calculation is mentioned in Section 2.1. A pointwise convolution (1 × 1 convolution) is then used as a channel mixer, which fuses information from all channels and pays more attention to features in the central region. The final framework retains the full training, annotation, and deployment pipelines of YOLOv5 to ensure the framework is a practical solution tailored to the constraints and needs of prestress wedge inspection in real factory environments.

## 3. Simulated Industrial Experimental Setup

To provide a solid technical basis for industrial validation of the applied lightweight framework in wedge detection, all tests adopted a unified experimental configuration, strictly in line with the low-cost technical conditions of small-to-medium prestress component factories (displayed in Table 1), and focused on solving on-site industrial pain points.

### 3.1. Dataset Construction

**Figure 4:**
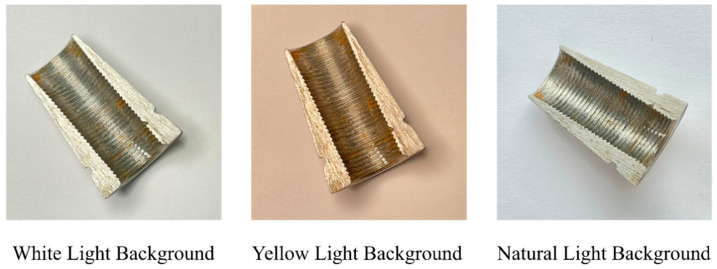
Various light backgrounds.

A total of 2000 defective wedge images were collected from 2024 retired batches (Lot No. 202403–202406) of Henan Great Wall Anchorage Manufacturing Pty. Ltd. (Kaifeng, China), utilizing a main camera lens of Sony IMX903 sensor (48 million pixels, 1/1.3 inch, ƒ/1.78 aperture, 2nd-generation sensor-shift OIS), with 7P lenses from Largan Precision (with sapphire glass coating). For p1 (incomplete thread), the missing length is 2–5 mm (15–30% of total thread length); p2 (scratch) has a depth of 0.1–0.3 mm and length of 3–8 mm; p3 (collision dent) has a diameter of 1–2 mm, consistent with GB/T 45575-2025 severity grades. p1, caused by machining tool wear, is at a high risk of strand slippage during tensioning, p2, caused by tool scratch, may weaken clamping force on strands, and p3, caused by cutter collision, does not satisfy the requirement of appearance standard. Sample images were captured under three real industrial lighting scenarios, covering main inspection locations, to address the most prominent on-site lighting variability, as shown in Figure 4. White light samples were collected at a fixed inspection station with 5000 K LED lights, 800 lux, and stable brightness, reflecting daily workshop inspection conditions. In an old workshop auxiliary area, yellow light samples were acquired under the condition of 3000 K aging fluorescent lights, 500 lux, uneven brightness with shadows, representing un-upgraded workshop environments in small factories. And natural light samples are used for workshops with sufficient lighting quantity under conditions of 4000–6500 K, 200–1000 lux, varying with weather. All images were directly captured with on-site issues like glare, shadow occlusion, and brightness fluctuation, aiming to ensure the model trained on this dataset can adapt to real industrial environments.

The dataset was randomly split into training (80%, 1600 images), validation (10%, 200 images), and test (10%, 200 images) subsets, with each subset maintaining the same proportion of flaw types and lighting conditions to avoid bias. To enhance the model robustness to on-site interference, random rotation with variable angles was adjusted to simulate wedge placement deviation on inspection conveyor movement, avoiding overfitting to ideal sample placement.

To provide a solid technical basis for industrial validation of the applied lightweight framework in wedge detection, all tests adopted a unified experimental configuration, strictly in line with the low-cost technical conditions of small-to-medium prestress component factories (displayed in Table 1), and focused on solving on-site industrial pain points.

### 3.2. Model Training for Industrial Adaptability

Images were annotated using LabelImg to generate bounding boxes by two senior factory inspectors with over 10 years of prestress component experience, strictly following GB/T 45575-2025 defect localization requirements. Then, training was designed to fit the technical capabilities of most prestress component factories and prioritize performance in industrial scenarios. Parameters were set as follows: batch size = 8 (adaptable to mid-range GPUs), momentum = 0.937 (standard for industrial object detection), image resolution = 640 × 640 (compatible with mainstream industrial cameras), weight decay = 0.0005 (prevents overfitting to small industrial datasets), and initial learning rate = 0.01 (gradually decays to 0.001, ensuring stable convergence). Each network was trained for 100 epochs.

### 3.3. Performance Evaluation Metrics

Metrics of precision, recall, F1-score, mAP@0.5, and mAP@0.5:0.95 were evaluated, with a focus on safety-critical indicators to meet mandatory requirements, e.g., recall for p1 flaw. Then, the model is able to avoid missed detections of critical flaws for engineering safety during industrial application. The deployment-oriented efficiency metrics, frames per second (FPS), inference time per frame (ms), and GFLOPs were tested to verify compatibility with industrial hardware. The final evaluations under three on-site lighting conditions focus on verifying the robustness to lighting variability, which not only validates the model algorithmic efficiency but also directly demonstrates the suitability for potential industrial deployment. Therefore, an industrial-representative dataset, an efficient factory-adaptable trained model, and a low-cost hardware-deployable configuration bridge the gap between practical deep learning research and new prestressing wedge inspection for subsequent industrial validations.

## 4. Results and Discussions

The applicability of the FasterNET-YOLOv5 framework was validated with a focus on detection stability, operational efficiency, and industrial adaptability in prestress wedge flaw inspection. The validation process focuses on the evaluation of consistent detection quality across illumination conditions, processing speed for real-time inspection, and satisfaction of practical engineering standards, aiming to provide direct reference for AI deployment in prestress anchorage quality control.

### 4.1. Detection Performance Metrics

The trained model was assessed using precision, recall, *F*1-score, and mAP@0.5 to evaluate detection accuracy and consistency. Precision, defined as the proportion of true positives (*TP*) among all predicted positives, can be denoted as shown in Equation (3).

(3) $$Precision=\frac{TP}{TP+FP}$$

where *FP* refers to false positives. It measures a model’s ability to minimize false inspections. High precision indicates a low probability of misclassifying non-targets as targets when the model predicts a target exists at a single confidence threshold and may be skewed by conservative predictions with quite a number of missed targets. In contrast, recall quantifies the model ability to minimize the missed targets and is critical for tasks where missed detections have severe consequences, as shown in Equation (4).

(4) $$Recall=\frac{TP}{TP+FN}$$

where *FN* represents false negative. Recall serves as a complement to be combined with the precision metric for providing a more holistic view. Then, *F*1-score, calculated as the harmonic mean of precision and recall (shown in Equation (5)), is selected to comprehensively reflect the optimal model metrics and balance the precision and recall performances. Focusing on binary/multi-class classification tasks, F1-score primarily evaluates per-class performance under fixed decision criteria.

(5) $$F1\;Score=\frac{2\times Precision\times Recall}{Precision+Recall}$$

Another key metric in model evaluation is mAP@0.5, which is defined by averaging the area under the precision–recall curve across all confidence thresholds of all classes, with IoU greater than 0.5 defining the overlap criterion between predicted and ground-truth bounding boxes. Distinct from *F*1-score, mAP@0.5 integrates precision–recall dynamics across all confidence thresholds, addresses the limitations of single-threshold metrics, reflects the model’s balance between lenient and strict thresholds, and ensures fairness in multi-class scenarios, thus effectively evaluating both localization and classification accuracy. mAP@0.5 is preferred for comprehensively assessing detection ability and prediction accuracy in multi-class, multi-target tasks to offer a holistic view of multi-class detection performance across thresholds and analyze model behavior from specific and global perspectives.

Successively, in validation, the detection metrics, precision, recall, *F*1-score, mAP@0.5, etc. from FasterNET-YOLOv5 models, are summarized in Table 2.

The FasterNET-YOLOv5 framework achieved a precision of 96.3%, a recall of 96.2%, and an mAP@0.5 of 96.5%. Minor variations between precision and recall correspond to the lightweight nature of FasterNET partial convolution, which filters redundant features for faster inference but slightly reduces sensitivity to extremely shallow flaws. In addition, although no specific standards have been established for prestress wedge flaw detection, China’s national standard GB/T 45575-2025 [29], scheduled for implementation in November 2025, has developed a comprehensive technical framework for industrial surface defect detection. Compared with the predecessor, GB/T 40659-2021 [30], GB/T 45575-2025 explicitly mandates technical upgrades based on deep neural networks, requiring a detection system with a mean average precision (mAP) of no less than 0.9, a flaw detection rate of at least 95%, and a missed detection rate of fewer than 5%, which verifies the framework capacity for reliable flaw detection in both coarse and fine thread regions of the wedge. Additionally, the data interfaces and model output formats are also compatible with the international standard ISO 13606-4:2021 [31]. Aligned with these practical industrial detection criteria, the FasterNET-YOLOv5 model proposed in this study with a precision of 96.3%, a recall of 96.2%, and an mAP@0.5 of 96.5%, outrates the benchmarks of industrial requirements. Moreover, prestress component factories focus more on no-missed detections of safety-critical flaws instead of how precise the framework is. Then, the decreased value in mAP@0.5–0.95 only points out the exact position of the flaw on the wedge, and therefore has no impact on safety or sorting.

### 4.2. Computational Efficiency

A comparison of efficiency metrics is listed in Table 3. The FasterNET-YOLOv5 framework achieves a substantial reduction to 45% in both floating-point operations (FLOPs) and parameter count, while preserving the fundamental capability of feature extraction. Notably, the significant reduction in the computational load of the backbone network remains dominant, driving the decrease in inference time, achieving 18% faster inference speed. Therefore, the FPS for FasterNET-YOLOv5’s inference time is 58 in comparison with 47 for YOLOv5, showing the evident speed increase by the lightweight backbone framework. Specifically, end-to-end FPS, serving as a meaningful indicator for potential engineering applications, covers the entire workflow from image reception to result output, including pre-processing, inference, and post-processing. When calculated using Equation (6), with a pre-processing time of 2.5 ms and NMS of 2.4 ms, FasterNET-YOLOv5 processes 45 images per second at a 18% faster speed than YOLOv5 with 38 processed images per second, due to the deviation of the inference time (17.2 ms vs. 21.1 ms).

(6) $$FPS=\frac{1}{Pre-process+inference+NMS}$$

Consequently, the reduced computational load and faster inference speed strongly demonstrate that the proposed FasterNET-YOLOv5 is manipulable within factory cycle times, supporting integration with continuous production workflows. In particular, the low parameter count and improved throughput facilitate the model integration into low-cost portable inspection devices or factory-line monitoring systems in future industrial studies.

Moreover, as shown in Table 3, the experimentally calculated retention ratio of parameters and Flops is 45%, inferring that the average group number average *P* ≈ 2.2. As discussed in Section 2.1, the grouping number *P* is dynamically arranged. Then, further investigation was conducted to understand the quantification of the average channel value. When replacing the backbone of YOLOv5s with FasterNET, the core principle is to retain the five-stage architecture, feature map dimensions, and channel counts, to ensure subsequent detection heads can receive features normally. Correspondingly, the standard convolutional layers within each stage are replaced with the partial convolution modules of FasterNET, with the specific parameters provided in Table 4.

To accurately reflect the actual impact of the grouping strategy on the overall network, it is necessary to adopt a weighted approach that incorporates the parameter ratio of each stage as the weight. In accordance with the design principles of the FasterNET block [26], the deep layers corresponding to Stages 3, 4, and 5 account for an extremely high proportion of the total parameters, with approximately 98.7% and *P* = 2 employed to maximize the retention of information. In contrast, the shallow layers of Stages 1 and 2 have a negligible impact, with their parameter proportion being only 1.3%, an insignificant contribution to the overall average; thus, *P* = 4 is used. Based on the grouping configurations of different stages, the specific weighted algorithm is presented in Equation (7).

(7) $$Average\;P=\sum \left(P_{i}\times {\mathrm{Ratio}}_{i}\right)$$

where $P_{i}$ represents the *P* value at different stages. Thus, the average grouping number can be calculated as *P* ≈ 2.04, which aligns closely with the experimental value (*P* ≈ 2.2), validating that channel count is the core determinant of the FasterNET inherent dynamic grouping strategy. A clear direction for optimizing the FasterNET block was achieved by refining the correlation between channel counts and grouping number *P* to enhance the precision of dynamic grouping. The strong structural compatibility after replacing the YOLOv5 backbone with FasterNET stems entirely from the consistent channel count variation patterns between the two architectures. Such consistency further suggests that the future optimization of *p* values, tailored to specific channel distributions in wedge flaw detection tasks, could further improve efficiency without disrupting integration with downstream detection modules.

### 4.3. Robustness Analyses

**Figure 5:**
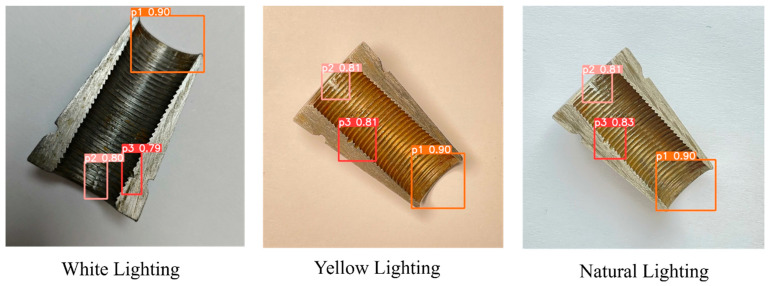
Exampled detection images from white yellow and natural lighting conditions.

To align with real-world prestress engineering scenarios, detection tests were conducted under three lighting conditions typical of prestress practice, white light (standard LED in modern wedge factories), yellow light (aging fluorescent lamps in old component workshops), and natural light (enough lighting quantity areas or storage yards), as displayed in Figure 5. The stable performance across these conditions directly solves the manual inspection errors under inconsistent lighting. More importantly, the results provide actionable guidelines for optimizing inspection setups to guarantee the structural safety.

**Figure 6:**
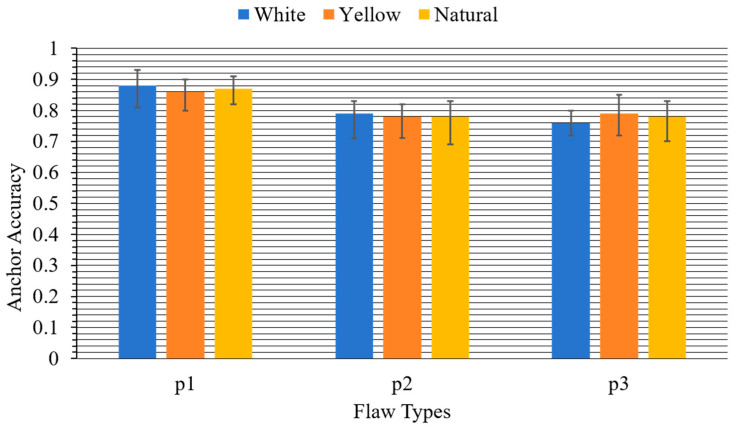
Visual detection accuracy under white yellow and natural lighting conditions.

Based on the collected detection results shown in the bar chart in Figure 6, for defect type p1, the average accuracy with deviations under white, yellow, and natural lighting conditions are $0.88_{-0.07}^{+0.05}$, $0.86_{-0.08}^{+0.04}$, and $0.87_{-0.04}^{+0.04}$, respectively. In contrast, the average accuracy values of p2 and p3 are lower than p1, $0.79_{-0.06}^{+0.04}$, $0.78_{-0.07}^{+0.04}$, $0.78_{-0.07}^{+0.06}$, and $0.76_{-0.05}^{+0.04}$, $0.79_{-0.09}^{+0.05}$ $0.78_{-0.08}^{+0.05}$, indicating that p1 may possess a regular size, distinct features, and high lighting sensitivity. The results also demonstrate that the anchor accuracy varies with both defect types and lighting conditions, and value deviations further reflect the stability of detection performance under different illumination environments. Notably, it is also shown that the anchor precision of detection results via detect.py under all lighting conditions exhibits a noticeable decline compared to the precision metrics from the validation set (shown in Table 5). The accuracy gap between val.py and detect.py reflects deliberate engineering trade-offs for on-site prestress wedge inspection. val.py prioritizes validation precision as the workflow uses letterbox padding to preserve aspect ratios, low confidence thresholds (0.001), relaxed NMS (0.6), and P-R curve integration to capture theoretical performance bounds. Contrastingly, detect.py optimizes for industrial practicality, setting higher confidence (0.25) and stricter NMS (0.45) to reduce false positives, which is critical for cost control. Though the detect operation lowers metrics, it ensures speed, cost-efficiency, and usability without compromising the detection of safety-critical flaws like p1 incomplete threads.

Specifically, the model exhibits varying robustness in identifying defects p1, p2, and p3 under white, yellow, and natural light, with distinct performance characteristics across defect types and illumination conditions. For p1, white light yields the highest accuracy 0.88 while natural light shows the most stable performance with error range ± 0.04. p2 performs best under white light (0.79) with moderate stability, though natural and yellow light introduce larger fluctuations with deviations of max +0.06 and min −0.07, respectively. p3 achieves the highest accuracy under yellow light (0.79) but with significant instability (min error of −0.09), whereas white light provides lower but more consistent results. Overall, white light offers the most balanced high precision across p1 and p2, natural light demonstrates the steadiest performance for p1, and yellow light is only advantageous for p3 at the cost of stability. Therefore, the fact that white light yields the most balanced detection performance suggests a straightforward adjustment in inspection environments that could significantly reduce missed detections of critical p1 flaws. Such results also indicate that inspection workshops can adopt standardized white light conditions to maximize flaw detection reliability. Such practical guidelines extend the contribution beyond algorithm design and provide immediate value to inspection practice in prestress engineering. Although the tests were confined to controlled datasets, transferable insights were acquired for real-world implementation, where illumination design is often overlooked in automated quality control systems.

### 4.4. Assistance of Mechanical Model Supported Inverse Methods for Flaw Detection

The current FasterNET-YOLOv5 framework excels at visual defect localization and classification but may face limitations in visually ambiguous situations, leading to potential false negatives. A mechanical model-informed inverse method can complement visual detection by providing mechanically meaningful validation for visually detected flaws and assisting in resolving visual ambiguities. Therefore, a simple mechanical wedge–strand–anchorage framework was deduced to check the engineering practices by linking the detections from deep learning.

#### 4.4.1. Mechanical Behavior of Wedge Gripping

**Figure 7:**
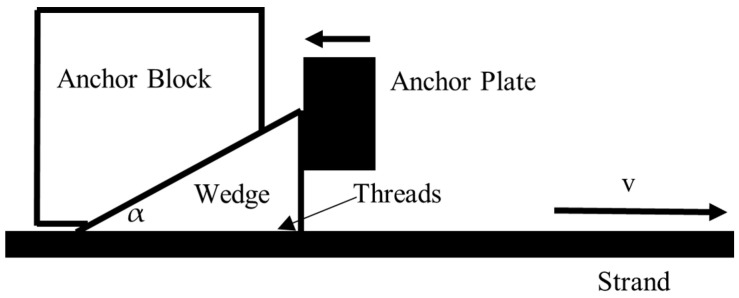
A simple mechanical wedge–strand–anchorage system.

Figure 7 shows the wedge gripping system. Following the strand pulled to the right, the anchor plate will push the wedge to the left by the jack. Then, the anchor block presses the wedge to grip the strand using threads. When tension reaches the maximum, the wedge fastens the strand to a fixed position. Therefore, under force balance condition, a simple mechanical model was derived to calculate the critical clamping force $F_{c}$ for preventing strand slippage:

(8) $$F_{c}=\frac{T}{\sin\alpha \;+\mu \cdot cos\;\alpha }$$

where T is the strand tension, μ is the friction coefficient between the wedge and strand, and α is the wedge half-taper. It is shown that the safety clamping force seriously correlates with the tension and the friction coefficient between wedge threads and strand.

#### 4.4.2. Mechanical Impact of Target Flaw Types

For the three safety-critical flaws in the acquired dataset, the model quantifies the specific mechanical effects, which are consistent with factory quality records and GB/T 45575-2025 defect severity criteria. p1 reduces the contact area between the wedge and strand by 20–30%, induced by thread missing length, which decreases $F_{c}$ by some percentage according to Equation (8). For example, a p1 flaw reducing $F_{c}$ by 30% may cause 2–3 mm strand slippage during tensioning [32], exceeding the GB/T 14370 [29] limit of 1 mm and leading to under-prestressing. In contrast, p2, the shallow scratch, introduces localized stress concentration at the scratch root. Although p2 does not immediately reduce $F_{c}$, it accelerates fatigue damage under cyclic loads (e.g., bridge vibration), shortening the wedge service life and causing catastrophic failure of the structure. p3 may create plastic deformation at the thread tip, misaligning the contact interface with the strand, and causing uneven stress distribution across the wedge. Hence, the mechanical model-based inverse method can map the visual flaw features to real engineering performance, enhancing detection reliability.

### 4.5. A Comparative Discussion with Newer YOLO Versions

A head-to-head comparison was conducted with mainstream lightweight variants (n-series) using the same experimental setup to highlight the model advantages, as shown in Table 6.

The proposed FasterNET-YOLOv5 model demonstrates superior performance across both lightweight YOLO variants and non-YOLO detectors, excelling in computational efficiency, inference speed, and detection accuracy. Compared to key lightweight YOLO models (YOLOv8n, v9-C, v10n–v12n), it achieves 18–23% lower FLOPs (7.1 G vs. 8.7–9.2 G), 3–8 FPS faster inference (58 FPS vs. 50–55 FPS), and a superior mAP@0.5 of 96.5% (0.5–2.3% higher than the 94.2–96.0% of comparators), alongside leading precision (96.3%) and recall (96.2%). Unlike the YOLO counterparts, which are optimized for specific scenarios such as flat surfaces (YOLOv10n), illumination adaptability (YOLOv12n), or small-defect detection (YOLOv9-C), FasterNET-YOLOv5 stands out in detecting the 3D geometry of prestress wedge products, addressing a critical gap in models tailored for planar scenarios. Critically, the advantage stems from the dynamic grouped PConv strategy: shallow layers (*P* = 4) filter redundant thread contour information, while deep layers (*P* = 2) retain critical flaw features on curved surfaces.

Against non-YOLO lightweight detectors like SSD-MobileNetV2 and EfficientDet-Lite3, FasterNET-YOLOv5 also balances efficiency and accuracy more effectively. SSD-MobileNetV2, despite achieving the lowest FLOPs (5.8 G) and highest speed (62 FPS), lags 6.5% in mAP@0.5 (90.0% vs. 96.5%); EfficientDet-Lite3, with its anchor-free design reducing hyperparameter complexity, still trails by 4.4% in mAP@0.5 (92.1% vs. 96.5%). These gaps arise because non-YOLO models, optimized for flat surfaces, fail to capture the complex textures and contours of 3D threaded structures. In contrast, FasterNET-YOLOv5 maintains a superior balance, with 7.1 G FLOPs (only 12% higher than SSD-MobileNetV2 but 18–23% lower than YOLOv8n–v12n) and 58 FPS, outperforming non-YOLO models by 4.4–6.5% in mAP@0.5 while retaining top-tier precision and recall. Complemented by 55% lower FLOPs than the original YOLOv5, stable illumination robustness, and balanced high performance, the current comparative context confirms the model’s uniqueness in overcoming industrial deployment constraints for threaded components.

Consequently, FasterNET-YOLOv5 is an optimal lightweight solution for prestress wedge flaw detection, boasting multi-dimensional core advantages, achieving top-tier accuracy (96.3% precision, 96.2% recall, 96.5% mAP@0.5), exceeding GB/T 45575-2025 national standard requirements, and delivering 100% recall for safety-critical incomplete thread (p1) flaws. With dynamic grouped PConv, the proposed model cuts FLOPs and parameters by 55% vs. the original YOLOv5, reaching 7.1 G FLOPs and 58 FPS (45 FPS end-to-end), enabling stable operation on low-cost embedded devices and solving industrial deployment pain points. Moreover, strong illumination robustness was also exhibited across white, yellow, and natural light, avoiding accuracy drops from lighting variations. Optimized for 3D threaded geometries, the FasterNET-YOLOv5 model outperforms mainstream YOLO variants (v8n–v12n) and non-YOLO models, balancing higher accuracy (0.5–2.3% better mAP@0.5) with lower computation and faster speed. Additionally, the integration of mechanical model inverse validation links the flaws with engineering safety metrics, while the modular design also supports production line integration and transfer to other 3D curved components, enhancing industrial practicality.

### 4.6. Engineering Applications and Future Directions

The FasterNET-YOLOv5 framework, while validated in controlled experiments, offers clear pathways for engineering application. Expressly, the lightweight design supports future deployment on embedded GPU devices and portable inspection kits, reducing reliance on manual checks. In addition, the approach provides a template for 3D surface inspection tasks involving threaded or curved geometries, where reflection and fine-scale detail remain challenging for conventional 2D methods. Particularly, integration with production line automation systems can be envisioned, where real-time flaw detection would feed directly into defect-sorting or quality grading modules, enabling continuous monitoring aligned with Industry 4.0 requirements.

More importantly, beyond prestressed wedges, the inspection of threaded and curved components remains a long-standing challenge in industrial quality control. Unlike flat steel plates or uniform weld seams, 3D geometries such as bolt threads, turbine blades, and gear surfaces introduce highly complex textures, curved reflections, and fine-scale defects that are often obscured under variable illumination. Conventional computer vision systems struggle with these conditions, as the feature distribution is both irregular and sensitive to environmental changes. The proposed FasterNET-YOLOv5 framework directly addresses the issues by demonstrating robustness to mixed lighting conditions and efficiency gains that make real-time inspection feasible. Although this research merely focused on prestress wedge flaws, the underlying methodology offers a transferable solution for general 3D surface inspection problems, highlighting the potential impact across aerospace, automotive, and energy sectors where defect detection in small but safety-critical components is equally urgent.

Despite the limitation of no hardware deployment or field trials in this study, the combination of high detection accuracy, robustness to illumination, and computational efficiency confirms the readiness of framework deployment. Future research will therefore prioritize extending the dataset to cover more diverse industrial scenarios, exploring integration with structured light or depth cameras for 3D flaw capture, and testing the framework in production line and on-site environments. These steps will strengthen the transition from algorithm development to fully automated, real-world quality assurance systems in prestressed concrete and beyond.

## 5. Conclusions

This study validated a FasterNET-enhanced YOLOv5 framework for intelligent detection of prestress wedge flaws under mixed illumination conditions. The framework achieved industrial-grade accuracy (precision 96.3%, recall 96.2%, mAP@0.5 96.5%) while reducing computational load by 55% and improving inference speed by 18% compared with YOLOv5s. The model’s robustness across illumination environments further supports its implementation in diverse workshop conditions. Moreover, the mechanical model-based inverse method further supplements the detections from machine vision with specific engineering reflections. The results also demonstrate that the framework is deployment-ready for real-time wedge inspection systems.

From an engineering standpoint, the FasterNET-YOLOv5 framework satisfies the performance criteria for industrial automation and provides a practical solution for the nondestructive evaluation of small, threaded components. Future work will extend dataset diversity, integrate 3D depth sensing for complex geometries, and deploy the framework on real prestress production lines to validate its long-term operational stability.

## Figures and Tables

**Table 1:** Test configurations.

Central Processing Unit (CPU)	Intel(R) N95 1.70 GHz
Graphic Processing Unit (GPU)	NVIDIA GeForce GTX1060 4 GB
Random Access Memory (RAM)	32.0 GB
Python Version	3.8.20
PyTorch Version	1.8.2
CUDA Version	10.2

**Table 2:** Detection accuracy of FasterNET-YOLOv5.

Model	Precision (%)	Recall (%)	F1-Score	mAP@0.5 (%)	mAP@0.5:0.95 (%)
FasterNET-YOLOv5	96.3	96.2	96.2	96.5	46.5

**Table 3:** Computational efficiency of YOLOv5 and FasterNET-YOLOv5.

Model	FLOPs (G)	Parameters (M)	Inference Time (ms/Frame)	FPS	End-To-End FPS
YOLOv5	15.8	7.02	21.1	47	38
FasterNET-YOLOv5	7.1	3.19	17.2	58	45

**Table 4:** Parameter ratios at all stages.

Stage	$C_{in}$	$C_{out}$	$k^{2}$$\times C_{in}$$\times C_{out}$ Parameters	Ratios
1	3	32	$k^{2}\;$ $\times \;3\;\times \;32=96\;k^{2}$	96/174,176 ≈ 0.1%
2	32	64	$k^{2}$ $\times \;32\;\times \;64=2048\;k^{2}$	2048/174,176 ≈ 1.2%
3	64	128	$k^{2}\;$ $\times \;64\;\times \;128=8192\;k^{2}$	8192/174,176 ≈ 4.7%
4	128	256	$k^{2}$ $\times \;128\;\times \;256=32,768\;k^{2}$	32,768/174,176 ≈ 18.8%
5	256	512	$k^{2}\;$ $\times \;256\;\times \;512=131,072\;k^{2}$	131,072/174,176 ≈ 75.2%

**Table 5:** Metrics of Different Flaws.

Flaw	Precision (%)	Recall (%)	mAP@0.5 (%)
p1	99.8	100	99.5
p2	98.0	97.3	98.5
P3	91.2	91.4	91.4

**Table 6:** Performance Comparisons of Various Light-weight Models.

Model	Precision (%)	Recall (%)	mAP@0.5 (%)	FLOPs (G)	FPS	Core Advantage
YOLOv6n	94.5	94.0	94.3	7.6	56	Balanced accuracy–efficiency
EfficientDet-Lite3	92.7	91.5	92.1	8.2	45	Anchor-free design reduces hyperparameter tuning complexity
SSD-MobileNetV2	90.3	89.7	90.0	5.8	62	Extremely low computational cost
RetinaNet-MobileNetV3-Large	93.1	92.6	92.8	7.9	51	Focal loss effectively addresses class imbalance in flaw detection
YOLOv8n	94.2	93.5	94.2	8.7	52	Balanced accuracy–efficiency
YOLOv9-C	95.1	94.8	95.1	9.2	50	Strong small-defect detection
YOLOv10n	95.5	95.0	95.5	8.9	54	Fast inference for flat surfaces
YOLOv11n	95.8	95.3	95.8	9.0	53	Enhanced feature fusion
YOLOv12n	96.0	95.5	96.0	8.8	55	Improved illumination adaptability
FasterNET-YOLOv5	96.3	96.2	96.5	7.1	58	Lightweight (55% lower FLOPs than YOLOv5) + high accuracy + stable illumination robustness

## Data Availability

The data presented in this study are available on request from the corresponding author.
